# Tissue concentration of vascular endothelial growth factor is not related to the depth of trophoblastic invasion in ampullary pregnancies—A pilot study

**DOI:** 10.3389/fphar.2022.989031

**Published:** 2022-10-20

**Authors:** Décio Roberto Kamio Teshima, Pedro Paulo Pereira, Rossana Pulcineli Vieira Francisco, Matheus Abelo De Oliveira, Regina Schulz, Leila Antonângelo, Fábio Roberto Cabar

**Affiliations:** ^1^ Department of Obstetrics and Gynecology, Faculty of Medicine, University of São Paulo, São Paulo, Brazil; ^2^ Hospital das Clínicas, Faculty of Medicine, University of São Paulo, São Paulo, Brazil; ^3^ Department of Pathology, Faculty of Medicine, University of São Paulo, São Paulo, Brazil; ^4^ Department of Clinical Pathology, Department of Pathology and Medical Research, Faculty of Medicine, University of São Paulo, São Paulo, Brazil

**Keywords:** first trimester hemorrhage, VEGF, vascular endothelial growth factor, ampullary pregnancy, trophoblast, pregnancy ectopic, tissue expression

## Abstract

**Introduction:** The factors that modulate trophoblastic invasion into the tubal wall remain uncertain. Moreover, it is known that the concentration of vascular endothelial growth factor (VEGF) is increased in cases of deeper trophoblastic invasion in the fallopian tubes.

**Objective:** This study aimed to assess if there is a correlation between VEGF tissue expression and the depth of trophoblastic infiltration into the tubal wall in patients with ampullary pregnancy.

**Methods:** A cross-sectional study was conducted in patients with a diagnosis of tubal pregnancy in the ampullary region who underwent salpingectomy. Inclusion criteria were spontaneously conceived singleton pregnancies, diagnosis of tubal pregnancy in the ampullary region, and radical surgical treatment. A lack of agreement regarding the location of the tubal pregnancy and impossibility of either anatomopathological or tissue VEGF analysis were the exclusion criteria. Histologically, trophoblastic invasion into the tubal wall was classified as grade I when limited to the tubal mucosa, grade II when it reached the muscle layer, and grade III when it comprised the full thickness of the tubal wall. A total of 42 patients fulfilled the inclusion criteria and were selected to participate in the study. Eight patients were excluded. After surgery, tissue VEGF expression was measured by immunohistochemistry and the point counting technique.

**Results:** Histological analysis revealed that eight patients had stage I tubal infiltration, seven had stage II, and 19 had stage III. The difference between the percentage of VEGF expression in the trophoblastic tissue was not significant in relation to the degree of trophoblastic invasion (*p* = 0.621) (ANOVA). Trophoblastic tissue VEGF showed no statistical difference for prediction of both degrees of trophoblastic invasion (univariate multinomial regression).

**Conclusion:** The depth of trophoblastic penetration into the tubal wall in ampullary pregnancies is not associated with tissue VEGF expression.

## 1 Introduction

Ectopic pregnancy (EP) is an obstetric complication in which the fertilized ovum implants outside the intrauterine cavity (Cunningham et al., 2022). The most common site of ectopic implantation is the fallopian tube (approximately 95% of cases), but other sites such as the ovaries and abdomen can be involved too (Tang et al., 2022). Seventy percent of fallopian tube pregnancies are located in the ampullary segment (Cunningham et al., 2022; Tang et al., 2022). EP is the most common cause of maternal mortality in the first trimester of pregnancy, justifying its clinical relevance (Creanga et al., 2017; Lisonkova et al., 2019). Its incidence has increased over the last decades and can be explained by the increased incidence of its risk factors, including pelvic inflammatory disease, the use of emergency contraceptive methods, and pregnancies conceived by assisted reproductive treatments (ACOG Practice Bulletin No, 2019).

Some potential predictors of trophoblastic invasion of the fallopian tubes in cases of EP, such as serum beta-hCG, transvaginal ultrasound, and maternal serum concentrations of vascular endothelial growth factor (VEGF), have been reported. Serum concentration of beta-hCG has high sensitivity and specificity in predicting trophoblastic tubal invasion and could be used to choose the best treatment for these patients (Cabar et al., 2010; Erol et al., 2015).

VEGF participates in the processes of implantation and placentation (Leung et al., 1989) and cellular VEGF production is increased in hypoxic conditions (Ladoux and Frelin, 1993). The implantation environment in the oviduct is very different from that of the well-vascularized endometrium, and production and secretion of VEGF seem to be elevated in EP in an attempt to acclimatize to an unfavorable environment (Zou et al., 2013; Zarezade et al., 2015).

The mechanisms that facilitate the trophoblastic invasion in the wall of the fallopian tube are unknown. We hypothesize that since there is a greater expression of VEGF in the EP implantation site, VEGF trophoblastic tissue concentration would be correlated with the depth of trophoblastic invasion into the wall of the oviduct.

Thus, the objective of the present study was to verify the correlation between trophoblastic tissue expression of VEGF in ampullary pregnancies and the depth of trophoblastic invasion into the tubal wall.

## 2 Materials and methods

A prospective study was conducted on patients with a diagnosis of tubal pregnancy in the ampullary region who underwent salpingectomy. Inclusion criteria were spontaneously conceived singleton pregnancies, diagnosis of tubal pregnancy in the ampullary region, and radical surgical treatment (salpingectomy). Cases in which there was no agreement regarding the location of the tubal pregnancy upon surgical description and histological analysis were excluded. Assessment of gestational age was made based on the last menstrual period. Institutional Review Board approval was obtained and informed consent was also obtained from each patient before participation in the study.

A total of 63 consecutive cases of EP were recorded during the study period. Of these, 21 patients were not included for different reasons: in one case, it was not possible to obtain the informed consent, and 20 patients were treated by a conservative approach (clinical or surgical). Forty-two patients fulfilled the inclusion criteria and were selected to participate in the study. Eight patients were excluded: in three patients, the tubal implantation site could not be identified, two showed no trophoblastic tissue in histological analysis, and in three, it was not possible to identify trophoblastic tissue VEGF.

To confirm the diagnosis, patients were routinely subjected to a serum beta-hCG determination; a transvaginal ultrasound was also performed. After diagnostic confirmation, if the patient could be enrolled in the protocol, informed consent was obtained.

After surgery, the fallopian tubes were immediately fixed in 10% formalin and sectioned serially for light-microscopic analysis. An average of 10 sections stained with hematoxylin-eosin was analyzed. To facilitate the identification of trophoblastic tissue invaded by the trophoblast, histological material was also stained with Masson’s trichrome to identify muscular fibers. Histological assessment was performed by a single well-experienced pathologist who was blinded to the clinical and laboratory characteristics of the patients.

Ampullary pregnancies were classified histologically according to the depth of trophoblastic infiltration into the tubal wall (Natale et al., 2003). In stage I, trophoblastic infiltration was limited to the tubal mucosa; in stage II, trophoblastic infiltration extended to the tubal muscularis; and in stage III, complete tubal wall infiltration with or without rupture of the serosa was observed. Immunohistochemical staining for human placental lactogen or cytokeratin 7 (hPL or CK7) was performed to identify trophoblastic cells and determine the depth of trophoblastic invasion in the tubal wall.

To analyze VEGF tissue expression, immunohistochemical reactions were performed on histological sections of the uterine tube fragments and studied by the biotin-streptavidin peroxidase method.

Histological sections of 3-μm thickness were obtained and collected on glass slides previously treated with 2% organo-silane adhesive solution (Sigma Aldrich Co., St. Louis, Missouri, United States). The slides were stained by hematoxylin-eosin for morphological analysis.

Next, the histological sections were deparaffinized in xylene at 60°C for 30 min, followed by two xylol baths at room temperature for 30 min and hydrated in a series of decreasing concentrations of ethanol, running water, and distilled water. After being washed in Tris-saline buffer (20 mM TBS and pH 7.4), the samples underwent the following protocol:

Rescue of the antigenic sites was performed with a steam cooker by immersing the slides in citrate (pH 6.0) for 1 min at 125°C. After this, the slides were washed with running water, distilled water, and PBS/Tween (Phosphate buffered saline-Twen® Tablets).

The slides were then incubated with monoclonal mouse anti-human VEGF (clone VG-1) (Santa Cruz, Biotechnology, Santa Cruz, CA, United States). Immunoglobulin G (IgG) from normal mice was used as control monoclonal antibodies. Tissues known to be positive were used as a positive control and omitted primary antibody as a negative control. (Positive control: Human tonsil, kidney, skin; negative control: omit primary antibody, isotype control, absorption control. The dilution used for all antibodies was 1: 800, carried out in a diluent solution, and applied over the cuts of the tissue. The slides were incubated overnight.

The slides were then washed in PBS/Tween and incubated in an oven at 37°C with Novolink Polymer secondary antibodies (Leica Biosystems Newcastle Ltd.).

After this step, the slides were washed in PBS/Tween and followed by the development of the chromogen 3, three diaminobenzidine (DAB) (Sigma Chemical Corporation code: D5637, St Louis, Missouri, United States). The slides were washed extensively in tap water and distilled water and counterstained with Harris hematoxylin (Merck, Darmstadt, Germany). Then, they were washed in running water and in distilled water, dehydrated, diaphanized, and mounted with resin for Entellan microscopy (Merck, Darmstadt, Germany).

**FIGURE 1:**
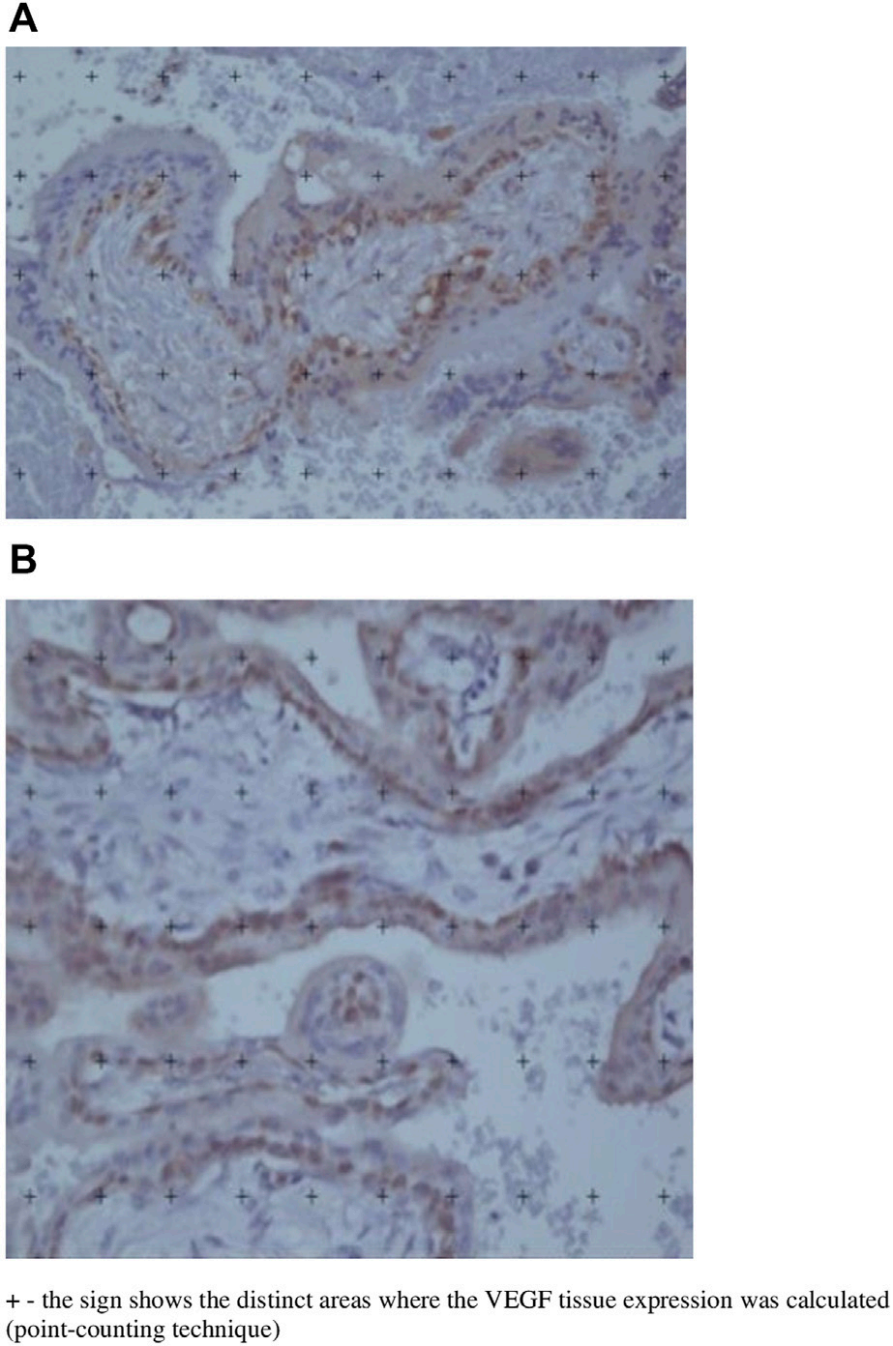
**(A)** and **(B)**: Histological images of two different fallopian tube segments affected by ectopic pregnancy after immunohistochemistry and microscopic view with tissue VEGF analysis using the point counting technique. (×400 magnification).

Evaluation of VEGF immunostaining in tubal tissue was performed by the stereological point-counting method (with modifications) based on Gundersen et al. (Gundersen et al., 1988) and using an image analysis system (Image-Pro Plus 6.0). (Fallopian tube tissues containing placental tissue were studied). Briefly, this system consists of a camera (Olympus Co., St Laurent, Quebec, Canada) coupled with a microscope (Olympus BX-51, Olympus Co., Tokyo, Japan), which captures images and sends them to a monitor with a scanning system (Oculus TCX, Coreco, Inc., St. Laurent, Quebec, Canada). A reticulum with 100 points was distributed over the orthogonally captured image. The assessment was performed by one observer, who was blinded, in 10 randomized fields of uterine tube tissue at increased magnification (×400). The percentage (*p*) of marked points in the reference compartment for VEGF was expressed according to the formula: *p* = (Pi × 100)/Pt; where Pi is the number of points that matches the positive marking by immunohistochemistry and Pt is the total number of points analyzed. The percentage (*p*) of VEGF was calculated from the sum of the results of all fields analyzed for each sample. (Figures 1–A,B).

Qualitative data were described using absolute and relative frequency (percentage) and were compared using Linear-by-Linear Association exact test.

For a summary of quantitative variables, median, minimum, maximum, mean and standard deviation (SD) were used. The difference between groups of trophoblastic invasion with respect of trophoblastic tissue VEGF was tested by parametric ANOVA test.

Multinomial logistic regression was used to compare the performance of the trophoblastic tissue VEGF expression associated to outcome the degree of trophoblastic invasion into the tubal wall. This analysis was applied to estimate the beta values and the odds ratios (OR) with 95% confidence interval (CI95%). The degree GI of trophoblastic invasion was considering the reference category.

*p* <.05 was considered statistically significant; all tests were two-tailed.

All statistical analyses were performed on a personal computer with the Statistics for Macintosh 22.0 (IBM Corp., Armonk, NY).

## 3 Results

The final sample consisted of 34 patients. The age of the women ranged from 11 to 40 years (29.8 ± 6.6 years), and there was no significant difference in mean maternal ages among the three histological groups (*p* = 0,706). Twenty four patients (70.4%) were Caucasian, and 14 (29.4%) were non-Caucasian. With respect to obstetric history, 18 patients (52.9%) were nulliparous and five (14.7%) had a history of EP in the contralateral fallopian tube. Histological analysis showed that eight patients (23.5%) had stage I tubal infiltration, 7 (20.6%) had stage II, and 19 (55.9%) had stage III. The gestational age ranged from 4 to 13.6 weeks (7.6 ± 2.1 week, and there was no significant difference in mean gestational ages among the three histological groups (*p* = 0,604).

According to ANOVA test, the difference between the percentage of trophoblastic tissue VEGF was not significant in relation to the degree of trophoblastic invasion (*p* = 0.621) (Table 1).

**TABLE 1:** Sociodemographic and clinical characteristics of patients associated to Trophoplastic invasion.

Covariates	Trophoblastic invasion			*p*
	GI (*n* = 8)	GII (*n* = 7)	GIII (*n* = 19)	
Age (average; SD)	30.6 (6.7)	26.3 (5.5)	30.8 (6.9)	0.297^1^
Ethnicity (*n*;%)				
White	6 (75.0)	4 (57.1)	14 (73.7)	0.774^2^
non white	2 (25.0)	3 (42.9)	5 (26.3)	
Marital status (*n*;%)				
with partner	6 (75.0)	1 (14.3)	5 (26.3)	0.053^2^
no partner	2 (25.0)	6 (85.7)	14 (73.7)	
Comorbidities (*n*;%)				
No	5 (62.5)	6 (85.7)	15 (78.9)	0.480^2^
Yes	3 (37.5)	1 (14.3)	4 (21.1)	
Previous delivery (*n*;%)				
No	1 (12.5)	5 (71.4)	12 (63.2)	0.041^2^
Yes	7 (87.5)	2 (28.6)	37 (36.8)	
Abortion (*n*;%)				
No	5 (62.5)	4 (57.1)	12 (63.2)	1.00^2^
Yes	3 (37.5)	3 (42.9)	7 (36.8)	
Ectopic pregnancy (*n*;%)				
No	6 (75.0)	7 (100)	16 (84.2)	0.783^2^
Yes	2 (25.0)	0 (0.0)	3 (15.8)	
VEGF tissue (average; SD)	20.8 (10.9)	19.7 (14.1)	16.5 (7.6)	0.547^1^

1.ANOVA; 2. Linear-by-Linear Association exact test.

Univariate multinomial regression analysis was performed and the variable trophoblastic tissue VEGF was included in order to compare its performance as a predictive factor of depth of trophoblastic invasion into oviduct wall. We observed that trophoblastic tissue VEGF showed no statistical difference for prediction of both degrees of trophoblastic invasion (Table 2).

**TABLE 2:** VEG tissue expression associated trophoplastic invasion, as determinated by univariate multinomial regression analysis.

Trophoblastic invasion		B(SE)	OR	95% CI		*p*
				Lower	Upper	
Group II	VEGF tissue (%)	−0.01 (0.05)	0,99	0,90	1,09	0,842
Group III	VEGF tissue (%)	−0.05 (0.04)	0,96	0,88	1,04	0,299
Reference category = Group I						

## 4 Discussion

In the past, the objective of EP treatment was to remove all trophoblastic tissue implanted in the uterine tube to preserve the patient’s life. Nowadays, due to the development of subsidiary tests, it has become possible to carry out more conservative treatments–both clinical and surgical.

Therefore, search for markers that can allow early diagnosis and selection of cases in which the tubal lesion is less deeper and does not compromise its function is of great interest.

In the studied population, 23.5% of patients had trophoblastic invasion limited to the mucosa, 20.6% had invasion limited to the muscle layer, and the remaining 55.9% had invasion throughout the thickness of the tubal wall. These findings are similar to the study by Cabar et al. (2006), which reported degrees of trophoblastic invasion as 27.6%, 28.6%, and 43.8%, respectively. Probably, the main explanation for this finding is the difficulty in early diagnosis of some cases of EP. There was any difference between groups regarding age, ethnicity, marital status, comorbidities, previous delivery, abortion nor ectopic pregnancies (Table 1).

It is known that VEGF expression is increased under unfavorable tissue conditions, including an hypoxic environment, such as occurs in the site where the embryo implants in the fallopian tube. In such cases, besides the increased levels of VEGF, an increase in the concentration of flt -1 receptor (VEGFR-2) is also observed, as shown by Vuorela et al. (Vuorela et al., 2000). Evans et al. (Evans et al., 1998) observed that the levels of VEGF and its receptor are elevated when choriocarcinoma cells are grown in a low-oxygen environment. Therefore, in addition to the increase in serum VEGF at the site of tubal implantation, its flt -1 receptor is also possibly increased, thereby lowering its free fraction.

Moreover, according to Evans et al. (1998), serum progesterone and beta-hCG concentrations are also increased in early stages of pregnancy and have an important relationship with the increase of VEGF, thus contributing to trophoblastic invasion. The authors stated that progesterone enhances VEGF production in epithelial cells of the retina and hCG increases VEGF in granulosa cells (Evans et al., 1998). Since the serum levels of progesterone and hCG are lower in EP, despite local hypoxia, VEGF production may remain unchanged due to the negative effect of the decreasing of other hormones.

Previous studies have shown that estrogen increases VEGF secretion in humans and animals (Torry et al., 1996; Torry and Torry, 1997). Similarly, with decreased serum levels of estrogen, progesterone, human chorionic gonadotropin, and possibly other cytokines and growth factors still unknown in EP could increase their concentrations *via* VEGF, but this conclusion is not supported by a statistically significant relationships.

We intended to associate the depth of trophoblastic invasion into the tubal wall and maternal trophoblastic tissue VEGF expression. From a study by Lam et al. (2004) showing higher VEGF concentrations in implantation sites of EP and according to the study of Cabar et al. (Cabar et al., 2010), who showed that serum VEGF is increased in cases of deeper trophoblastic invasion, our initial hypothesis was that higher trophoblastic tissue VEGF expression could lead to a deeper invasion of trophoblastic cells into the wall of the uterine tube. We believed that this information could bring better understanding about the factors that facilitate trophoblastic invasion into the fallopian tube.

However, the percentage of trophoblastic tissue VEGF was similar in the three degrees of trophoblastic invasion (*p* = 0.621) and did not attain statistical difference. This finding suggests that VEGF at the site of tubal implantation appears not to be related to trophoblastic invasion, and perhaps others cytokines and other still unknown growth factors may contribute to this process. (Table 2) Zarezade et al. (2015) found similar results when investigating the expression of VEGF mRNA and VEGF receptor 1 (VEGFR1) and 2 (VEGFR2) in women with EP compared to women who underwent hysterectomy. The authors concluded that the expression of VEGF and its receptors is lower in women with EP compared to women with normal tubes.

Through this study, we tried to understand the reason why trophoblastic tissue invasion finds favorable conditions in some cases and reaches the deepest layers of the fallopian tube. The mechanism that explains factors determining the depth of tissue invasion in ectopic pregnancies remains unknown. We believe this information is important as it could help to understand the histopathological mechanism involved in this process, providing new fronts for the diagnosis and treatment of this pregnancy complication. To our knowledge, this information is not yet available in the literature.

As a conclusion, we found that, despite previous studies indicating that serum VEGF is related to trophoblast invasion in the tubal wall in EPs, the present investigation did not find this association between tissue expression of this molecule and the depth of trophoblast invasion in the tubal wall in EPs. The tissue expression of VEGF at EP implantation site may not be primarily responsible for local modifications conducive to the development of a trophoblast.

The principal limitation of the study was the sample size, since we did not have a great number of cases. We believe that further studies, with larger sample sizes, should be carried out in order to better understand this histopathological process.

## Data Availability

The datasets presented in this article are not readily available. All the data generated in this study are anonymous and there is no way to individualize or identify patients. In Brazil, by law, this data cannot be provided to third parties. Further inquiries can be sent to the corresponding author FC, fabio.cabar@hc.fm.usp.br.
